# Genome-wide characterization of non-reference transposable element insertion polymorphisms reveals genetic diversity in tropical and temperate maize

**DOI:** 10.1186/s12864-017-4103-x

**Published:** 2017-09-06

**Authors:** Xianjun Lai, James C. Schnable, Zhengqiao Liao, Jie Xu, Gengyun Zhang, Chuan Li, Erliang Hu, Tingzhao Rong, Yunbi Xu, Yanli Lu

**Affiliations:** 1 Maize Research Institute, Sichuan Agricultural University, Wenjiang, Sichuan 611130 China; 2 Institute of Crop Science, Chinese Academy of Agricultural Sciences, Haidian, Beijing, 100081 China; 3 Bejing Genomics Institute (BGI)-Shenzhen, Shenzhen, 518083 China; 4 Department of Agronomy and Horticulture, University of Nebraska-Lincoln, Lincoln, NE 68583 USA; 5 International Maize and Wheat Improvement Center (CIMMYT), El Batan, Texcoco, CP 56130 México

**Keywords:** Adaptation, Genetic recombination, GWAS, Maize, Transposable elements, Non-redundant TEs (NRTE)

## Abstract

**Background:**

Maize was originally domesticated in a tropical environment but is now widely cultivated at temperate latitudes. Temperate and tropical maize populations have diverged both genotypically and phenotypically. Tropical maize lines grown in temperate environments usually exhibit delayed flowering, pollination, and seed set, which reduces their grain yield relative to temperate adapted maize lines. One potential mechanism by which temperate maize may have adapted to a new environment is novel transposable element insertions, which can influence gene regulation. Recent advances in sequencing technology have made it possible to study variation in transposon content and insertion location in large sets of maize lines.

**Results:**

In total, 274,408 non-redundant TEs (NRTEs) were identified using resequencing data generated from 83 maize inbred lines. The locations of DNA TEs and copia-superfamily retrotransposons showed significant positive correlations with gene density and genetic recombination rates, whereas gypsy-superfamily retrotransposons showed a negative correlation with these two parameters. Compared to tropical maize, temperate maize had fewer unique NRTEs but higher insertion frequency, lower background recombination rates, and higher linkage disequilibrium, with more NRTEs close to flowering and stress-related genes in the genome. Association mapping demonstrated that the presence/absence of 48 NRTEs was associated with flowering time and that expression of neighboring genes differed between haplotypes where a NRTE was present or absent.

**Conclusions:**

This study suggests that NRTEs may have played an important role in creating the variation in gene regulation that enabled the rapid adaptation of maize to diverse environments.

**Electronic supplementary material:**

The online version of this article (10.1186/s12864-017-4103-x) contains supplementary material, which is available to authorized users.

## Background

Maize (*Zea mays ssp. mays*) was domesticated approximately 9000–10,000 years ago from teosinte (*Zea mays ssp. parviglumis*), an indigenous grass species native to the tropical lowlands of southwest Mexico (17° ~28° N) [1, 2]. The divergence of tropical and temperate maize began when maize spread from its tropical origin south to Peru and Chile (10° N–29° S) approximately 3800–7000 years ago, and north to the temperate northern and eastern latitudes of North America (32°–49° N) approximately 3400–6700 years ago [3–5]. The range of maize expanded even further in the last 400–500 years when it reached East Asia, Europe, and Africa [5].

The wide migration of maize cultivation has placed strong pressure on maize populations to adapt to changing geographic and environmental conditions. This selective pressure could act on either standing genetic variation or novel allelic variation to create change in phenotype. Subsequent selective breeding to develop new improved varieties, including better traits in yield, quality, or adaptation, gradually altered maize further to meet the needs of farmers in different regions [6, 7]. Researchers have sought for decades to identify the genes associated with phenotypic variation and climate adaptation through association analysis by using genetic markers including SSRs, InDels, and SNPs [5, 7]. More recent transposable elements (TEs) of insertion polymorphisms have been used for both analysis of population structure and demographic history in maize, as well as association analysis with various traits of interest [8].

TE polymorphisms near or in genes are sometimes associated with dramatic phenotypic changes in a variety of organisms. These changes can result from either insertion into and disruption of the coding sequence of individual genes, altering gene regulation, or serving as templates for large-scale genomic rearrangements [9–11]. Moreover, TEs are often induced by stresses to the host plant [12, 13]; for example, the rice transposon *mPing* can be activated in response to cold and salt stresses [14], and the *Arabidopsis* retrotransposon *ONSEN* can be transcriptionally activated by heat stress [15]. Also, these TE-induced mutations have frequently been associated with environmental adaptation [12, 13]. In soybean, the disruption caused by a TE in the gene *GmphyA2* encoding phytochrome A has been associated with the adaptation of this species to high latitudes [11]. In *Arabidopsis*, the genes *FAR1* and *FHY3*, which developed from an ancient *Mutator*-like transposase, may have contributed to the adaptation of *Arabidopsis* to changing light environments [16].

Some researchers have argued that because plants live in a dynamic environment, selection based on standing genomic variation may be too slow to allow the plant to adapt to rapid changes in their environments [17, 18]. Therefore, TE-mediated insertions and mutations likely contribute to adaptation to major environmental changes, such as maize’s adaptation to temperate zones. Transposon insertion alleles have been shown to produce phenotypic variation in maize. A *Hopscotch* TE inserted in the regulatory region of *teosinte branched1* (*tb1*) gene was shown to enhance gene expression and repress branch growth [19]. A CACTA-like TE insertion was shown to repress *ZmCCT* expression, resulting in a reduction of photoperiod sensitivity and contributing to the adaptation of maize to long-day environments [20]. A miniature transposon (MITE) insertion into a conserved noncoding sequence upstream of a known flowing-time regulation gene *Vgt1* in maize was highly associated with early flowering [21]. These studies, which focused on individual TE insertions in maize, demonstrate the potential of TE insertion polymorphisms to directly affect phenotype. TEs comprise more than 85% of maize genome and play a large role in intraspecific genomic variation. For example, between 25% and 84% of the differences among modern maize inbred lines are the result of TE insertion polymorphisms; however, little is known about their contribution to phenotypic and genetic variation, particularly the roles they played in the temperate climate adaptation [8, 22]. TEs as molecular markers could also be incorporated into genome-wide association mapping studies.

Here, we conduct a genome-wide analysis of unique non-reference TE (NRTE) insertions in temperate and tropical maize lines. The goal of this study was to compare haplotype variation between tropical and temperate maize lines and to identify potentially adaptive insertions linked to changes in flowering-related traits by performing an association study on a large panel of inbred lines. NRTEs not present in the reference genome were found to have a bias toward insertion near genes, but also with a preference for avoiding coding regions. NRTEs unique to temperate maize germplasm were enriched in insertions adjacent to genes annotated as involved in stress response pathways, suggesting that NRTEs show distinct characteristics relative to the overall population of TEs in the maize genome [23]. Overall, our results suggest that TE-induced variation in environment adaptation–related phenotypes may have been an important factor in the adaptation of maize to temperate environments.

## Results

### Identification and genomic distribution of NRTE insertion in tropical and temperate maize lines

**Fig. 1:**
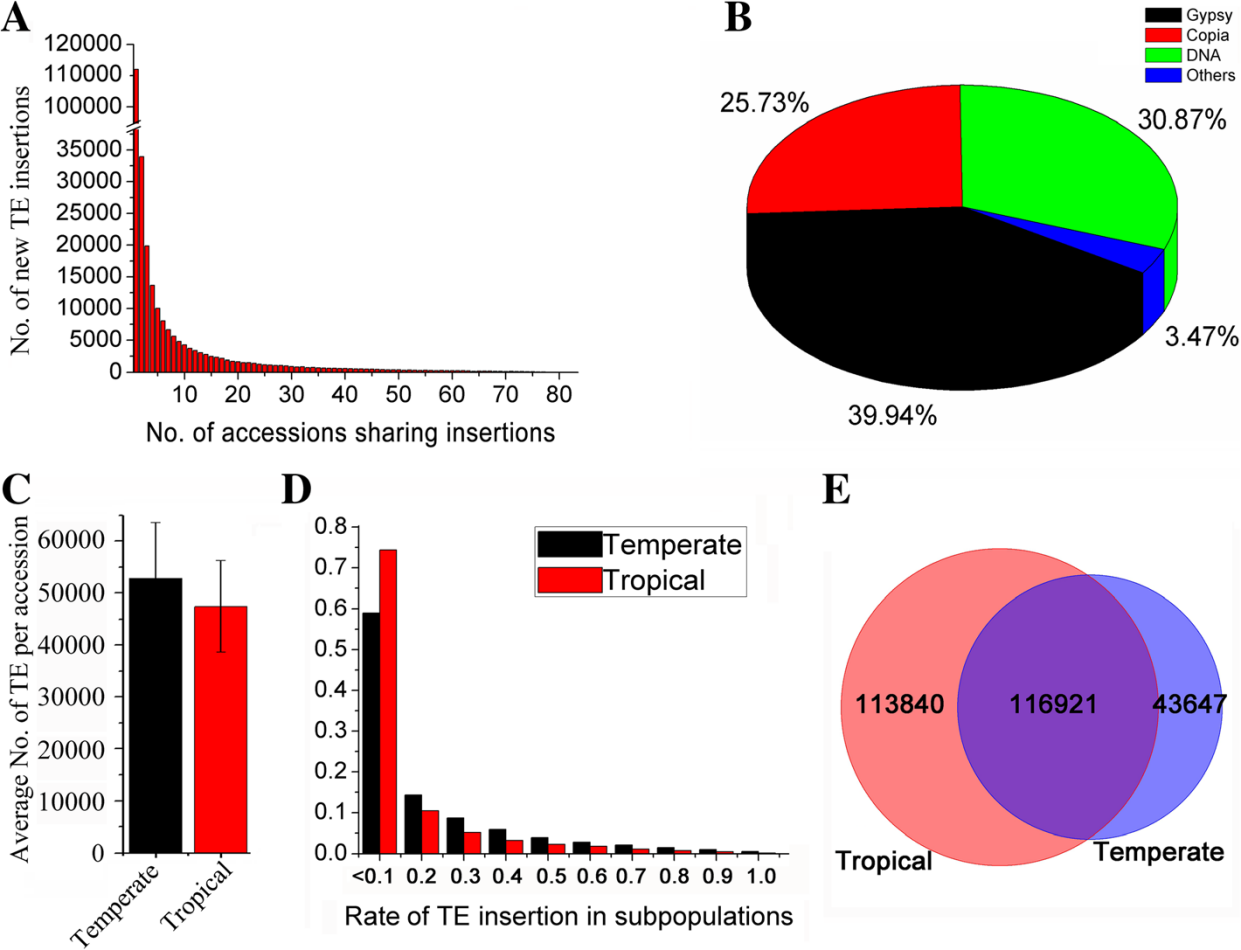
Scanning of NRTE insertions in 83 maize *inbred lines* according to the B73 reference genome. **a** Number of NRTE insertions present in one or shared by two or multiple inbred lines. **b** Proportions of different categories of NRTE in the 83 maize i*nbred lines*. **c**-**d**) Average number and frequency of NRTE insertions in temperate and tropical *maize lines*. Error bars represent the standard deviation. **e** Numbers of NRTE present in temperate (*blue*), tropical (*orange*), and shared by both (overlap)

Resequencing data from 83 maize inbred lines, including 31 temperate and 52 tropical maize lines, were screened for TE insertion sites (Additional file 1: Table S1), following the methods described by Tian et al. [24] and Ewing and Kazazian [25]. This semi-automated bioinformatic pipeline includes several steps: 1) establishing dataset of maize TE edge sequences, 2) detecting TE-flanking sequence junction sites from resequencing reads, 3) filtering of TE-edge fragments shared by the reference genome, 4) identifying the unique non-TE portions of TE-edge fragments, and 5) computing the frequency of NRTE insertions for temperate and tropical maize lines (see Methods). In total, 274,408 NRTE insertions mapped to unique sites in the B73 reference genome were identified. Among all NRTE insertions, 111,967 (40.8%) were detected in a single maize line, 33,943 (12.4%) in only two maize lines, and 128,498 (46.8%) in three or more maize lines (Fig. 1a). These NRTE insertions then were classified based on transposon class. Among the approximately quarter million NRTEs, 30.87% were DNA TEs, 65.67% were LTR-RTs (including 39.94% Gypsy-superfamily and 25.73% Copia-superfamily), and 3.47% were other TE classes (Fig. 1b). Among NRTEs inserted into annotated genes, NRTE insertions were most frequent in UTRs, followed by introns and exons (Additional file 1: Table S2; Additional file 2: Figure S1). This result is consistent with expectations given that insertions into exons could directly lead to deleterious genetic mutations and gene disruption, such as the disruption of coding sequences or predicted binding sites, introducing frame-shifts, etc. In contrast, insertions into UTRs are often silent, and when they produce phenotypes, the altered phenotypes may involve altered expression patterns that result in deleterious or neutral effects, or that provide adaptive benefits.

**Fig. 2:**
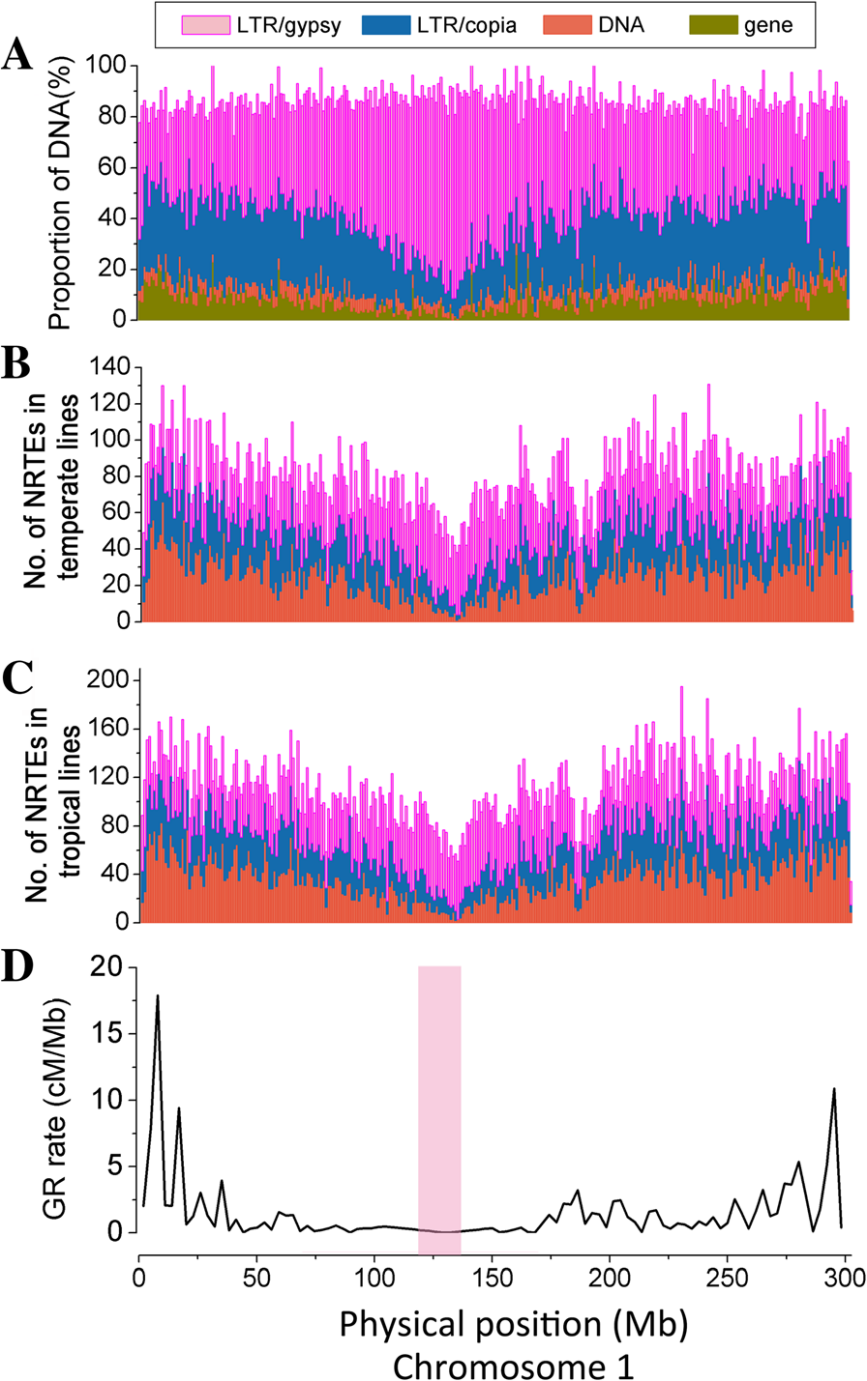
Characterization of transposable element (TE) distribution and genomic features along chromosome 1. **a** Distribution of TEs and genes in the B73 reference genome, the remaining proportion to reach 100% is intergenic space. **b**-**c** Distribution of non-redundant TEs (NRTEs) in the temperate and tropical maize lines. **d** Variation in genetic recombination (GR) rates along the chromosome in the B73 reference genome. The pink highlighted area defines the pericentromeric region on the chromosome. cM: centimorgans

Temperate maize lines had higher average numbers of NRTE insertions than tropical maize lines (52,858 versus 47,494; Fig. 1c) (t-test, *p* = 0.08). Temperate and tropical lines showed significant differences in the chromosomal distribution of NRTE insertions relative to transposons present in the maize reference genome. In the reference genome, DNA-TEs were distributed across 10 chromosomes without preference, except for a slight decrease in centromeric regions, whereas LTR-RT density in centromeric regions was almost 20% higher than that in chromosome arms. The two LTR-RT subclasses showed opposite distribution patterns in the reference genome. LTR/*Copia* is mainly distributed in chromosomal arms, whereas the LTR/*Gypsy* had the highest density in centromeric regions (Additional file 1: Table S3; Fig. 2a; Additional file 3: Figure S2a). These data were consistent with previous results [26]. However, NRTEs tended to have higher densities in chromosomal arms than in pericentromeric regions in temperate and tropical lines (Fig. 2b-c; Additional file 3: Figure S2b-c), and the observed density of each type of NRTEs in different chromosome regions had a significantly positive correlation between temperate and tropical lines, with correlation coefficients ranging from 0.81 (LTR-RTs) to 0.95 (DNA TEs) (Table 1). Intriguingly, a significant negative correlation for LTR-RT density across chromosomes was found between insertions present in the reference genome and NRTEs in tropical/temperate maize lines (Table 1).

**Table 1:** Correlation of NRTEs in their sequencing maize lines with TEs in B73

Features Compared	Whole Chromosomes		Chromosomal Arms	
	r^a^	*P*-value	r^a^	*P*-value
Densities of LTR-RTs: TEM versus TST	0.809	2.20 × 10^−16^	0.813	2.20 × 10^−16^
Densities of Gypsy-superfamily: TEM versus TST	0.811	2.20 × 10^−16^	0.814	2.20 × 10^−16^
Densities of Copia-superfamily: TEM versus TST	0.861	2.20 × 10^−16^	0.845	2.20 × 10^−16^
Densities of DNA TEs: TEM versus TST	0.947	2.20 × 10^−16^	0.942	2.20 × 10^−16^
Densities of LTR-RTs in TEM versus accumulated LTR-RTs in B73 genome	−0.067	2.37 × 10^−03^	−0.037	1.17 × 10^−01^
Densities of Gypsy-superfamily in TEM versus accumulated Gypsy-superfamily in B73 genome	0.374	2.20 × 10^−16^	0.352	2.20 × 10^−16^
Densities of Copia-superfamily in TEM versus accumulated Copia-superfamily in B73 genome	0.514	2.20 × 10^−16^	0.438	2.20 × 10^−16^
Densities of DNA TEs in TEM versus accumulated DNA TEs DNA in B73 genome	0.301	2.20 × 10^−16^	0.257	2.20 × 10^−16^
Densities of LTR-RTs in TST versus accumulated LTR-RTs in B73 genome	−0.130	3.83 × 10^−09^	−0.099	2.29 × 10^−05^
Densities of Gypsy-superfamily in TST versus accumulated Gypsy-superfamily in B73 genome	0.348	2.20 × 10^−16^	0.332	2.20 × 10^−16^
Densities of Copia-superfamily in TST versus accumulated Copia-superfamily in B73 genome	0.526	2.20 × 10^−16^	0.449	2.20 × 10^−16^
Densities of DNA TEs in TST versus accumulated DNA TEs in B73 genome	0.309	2.20 × 10^−16^	0.264	2.20 × 10^−16^

TEM means the resequencing temperate maize lines; TST means the resequencing tropical/subtropical maize lines

^a^Pearson correlation coefficient. TE: transposable elements; NRTEs: non-redundant TEs

### Validation of NRTE insertions and their association with gene expression

**Fig. 3:**
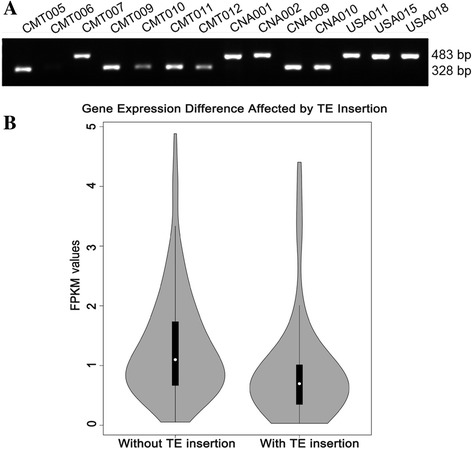
Validation of the presence or absence of NRTEs and their effect on gene expression. **a** PCR-based validation of a NRTE insertion in the resequencing *maize lines*. The different size of the bands indicated different accessions with or without NRTEs insertions in different accessions. NRTEs: non-reference transposable elements. **b** FPKM values of gene expression with or without TE insertion in 368 maize *inbred lines*. FPKM: fragment per kilobase of exon model per million mapped reads

To evaluate the NRTE identification pipeline, a PCR-based method was used to validate the NRTE insertions identified in silico by using maize resequencing data. Four NRTEs with high frequency in tropical or temperate maize were selected for validation. PCR amplification was performed on 75 maize lines utilizing DNA from the same seed sources used for whole genome resequencing (Additional file 1: Table S1), and then the PCR products were sequenced (Fig. 3a). A 155-bp DNA TE named *DTM-4-225,713,639*, inserted between GRMZM2G018673 and GRMZM2G702831; a 138-bp DNA TE named *DTM-2-4,101,264*; and other two NRTEs (which have similar results and are not shown) were amplified (Additional file 1: Table S4). The allele frequency for the insertion allele *DTM-4-225,713,639* was 62.1% and 15.2% in the temperate and tropical maize lines, respectively. These results were close to the expected values (71.0% and 5.80%) obtained by genome-wide scanning. In 85.3% (64/75) of cases, the result from the PCR experiment agreed with the computational prediction from the resequencing data. Among the remaining cases, 10.7% (8/75) lines showed an insertion based on PCR but not based on resequencing, and 4% (3/75) showed an insertion based on resequencing but not PCR. *DTM-2-4,101,264* insertion frequencies were 25.9% and 80.0% in temperate and tropical maize, respectively, which was also consistent with genome-wide scanning results (19.4% and 76.9%). In 89.3% (67/75) of cases, the result from the PCR experiment agreed with the computational prediction from the resequencing data. Among the remaining cases, 9.3% (7/75) lines showed an insertion based on PCR but not based on resequencing, and 1.3% (1/75) showed an insertion based on resequencing but not PCR.

In an enlarged association population of 368 maize inbreds, the frequencies of the transposon’s presence or absence in PCR experiments were 40.9% and 18.5% for *DTM-4-225,713,639*, and 44.8% and 65.75% for *DTM-2-4,101,264*, in temperate and tropical maize lines, respectively. Subsequently, the correlation between the presence of NRTE insertion and gene expression was examined through association mapping using the expression values of 28,850 genes collected for the 368 maize inbred lines [27]. In total, 21 genes were significantly associated with NRTE *DTM-4-225,713,639* at the Bonferroni-corrected threshold of *p* ≤ 3.47 × 10^−5^ *,* including the significant gene GRMZM2G018707 (*p* = 4.19 × 10^−06^), located 25 kb upstream of *DTM-4-225,713,639*. t-test of the fragment FPKM (per kilobase of exon model per million mapped reads) values for GRMZM2G018707 indicates that *DTM-4-225,713,639* significantly (*p* < 0.001) affected the gene expression, with an average expression value of 0.898 with the TE insertion, compared to 1.33 without the insertion, suggesting that GRMZM2G018707 gene expression was inhibited by the TE insertion (Fig. 3b).

### Association of NRTEs with gene density and genetic recombination rate

A previous study showed a strong association of gene density and genetic recombination (GR) rate [28]. The relationship between TE insertions and GR rates in the B73 reference genome first was quantified using SSR markers [28, 29]. Genetic recombination rates were higher in chromosomal arms than in centromeric regions (Fig. 2d; Additional file 3: Figure S2d), which are heterochromatin regions with low gene content and restrained GR rates [22]. Significantly negative correlations were observed between gene contents/GR rates and total TEs, LTR-RTs, or LTR/*gypsy* on all 10 chromosomes in B73 (Additional file 1: Table S5). However, DNA TEs and LTR/*Copia* showe0d statistically significant positive correlations with gene content and GR rate (Additional file 1: Table S5; Fig. 2a; Additional file 3: Figure S2a).

Next, the correlations between NRTE contents in tropical/temperate maize lines and gene densities/GR rates in B73 were estimated (Table 2). In agreement with the correlations described above, LTR/*gypsy* density across chromosomes in tropical/temperate lines showed a significantly negative correlation with gene density and GR rate in B73, whereas LTR/*copia* and DNA TEs showed significantly positive correlations with each other. LTR/*gypsy* insertions and gene density showed a stronger correlation, whereas other NRTEs had a weaker correlation in tropical lines than in temperate lines (Table 2). Moreover, correlation coefficients of GR rates with NRTE density in the resequencing maize genomes decreased for the LTR/*gypsy*, slightly increased for the LTR/*copia*, and greatly increased for DNA TEs, compared to the correlations identified with TEs in the B73 reference genome. Gene density had a lower negative correlation with the LTR/*gypsy*, lower positive correlation with the LTR/*copia*, and higher positive correlation with DNA TEs, compared to B73 TEs (Tables 2; Additional file 1: Table S5).

**Table 2:** Correlation of NRTEs in temperate and tropical maize lines with genome features in the reference genome

Features Compared	Whole Chromosomes		Chromosomal Arms	
	r^a^	*P*-value	r^a^	*P*-value
LTR-RTs density in TEM versus GR rates	−0.069	3.89 × 10^−02^	−0.120	7.62 × 10^−04^
Gypsy-superfamily density in TEM versus GR rates	−0.307	2.20 × 10^−16^	−0.314	2.20 × 10^−16^
Copia-superfamily density in TEM versus GR rates	0.250	3.84 × 10^−14^	0.172	1.64 × 10^−06^
DNA TEs density in TEM versus GR rates	0.562	2.20 × 10^−16^	0.529	2.20 × 10^−16^
LTR-RTs density in TST versus GR rates	−0.041	2.22 × 10^−01^	0.093	1.02 × 10^−02^
Gypsy-superfamily density in TST versus GR rates	−0.290	2.20 × 10^−16^	−0.298	2.20 × 10^−16^
Copia-superfamily density in TST versus GR rates	0.285	2.20 × 10^−16^	0.212	2.80 × 10^−09^
DNA TEs density in TST versus GR rates	0.596	2.20 × 10^−16^	0.568	2.20 × 10^−16^
LTR-RTs density in TEM versus gene density	0.044	4.35 × 10^−02^	0.011	6.46 × 10^−01^
Gypsy-superfamily density in TEM versus gene density	−0.379	2.20 × 10^−16^	−0.360	2.20 × 10^−16^
Copia-superfamily density in TEM versus gene density	0.479	2.20 × 10^−16^	0.420	2.20 × 10^−16^
DNA TEs density in TEM versus gene density	0.787	2.20 × 10^−16^	0.767	2.20 × 10^−16^
LTR-RTs density in TST versus gene density	0.096	1.28 × 10^−05^	0.060	9.95 × 10^−03^
Gypsy-superfamily density in TST versus gene density	−0.358	2.20 × 10^−16^	−0.340	2.20 × 10^−16^
Copia-superfamily density in TST versus gene density	0.534	2.20 × 10^−16^	0.480	2.20 × 10^−16^
DNA TEs density in TST versus gene density	0.819	2.20 × 10^−16^	0.801	2.20 × 10^−16^

TEM means the resequencing temperate maize lines; TST means the resequencing tropical/subtropical maize lines

^a^Pearson correlation coefficient. NRTEs: non-redundant transposable elements

**Fig. 4:**
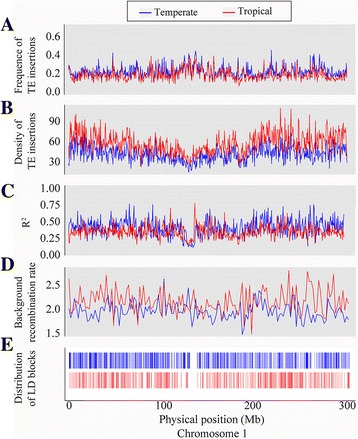
Relationship among NRTEs, BR rates, and LD patterns on chromosome 1 in temperate and tropical maize lines. **a**-**b** Frequencies and densities of NRTEs on chromosome 1 in temperate and tropical *maize lines*. **c**-**d** LD patterns revealed by R^2^ and BR rate in temperate and tropical *maize lines*. **e** Distribution differences of LD blocks between temperate and tropical *maize lines*. *Green* and *red boxes* indicate LD blocks (> 5 kb) in temperate and tropical maize lines, respectively. NRTE: non-redundant transposable element; LD: linkage disequilibrium; BR: background recombination

Genetic diversity in diverse maize germplasm was associated with linkage disequilibrium (LD) decay, and tropical maize germplasm has generally shown a smaller LD decay distance than temperate maize germplasm, suggesting higher genetic diversity in tropical maize, which is consistent with the demographic history of maize [30]. To determine whether the NRTEs effected the genetic diversity in temperate/tropical lines, we then analyzed the relationships among NRTE frequency, LD, and background recombination (BR) rate in temperate and tropical maize lines. The result showed that low-frequency NRTE insertions (< 0.1) were more commonly observed in tropical (~70%) than in temperate lines (~55%). However, intermediate-frequency NRTE insertions (0.1–0.5) were ~1.62 times more common in temperate than in tropical lines, and this ratio increased to ~1.86 times for high-frequency NRTE insertions (> 0.5) (Fig. 1d). In order to correct for any bias caused by the different numbers of tropical and temperate lines used in this analysis, 31 lines were randomly sampled from the 52 tropical lines, which produced equivalent results. Hence, the total number of unique NRTE insertions was lower in temperate lines than in tropical lines (43,647 versus 113,840; Fig. 1e). The average frequencies and densities of NRTEs across chromosomes were 0.196 per site and 38.65 per Mb in temperate lines, compared to 0.168 per site and 55.58 per Mb in tropical lines (Fig. 4a, Additional file 4: Figure S3a). Tropical maize had higher TE density and lower TE frequency than temperate maize (Fig. 4b, Additional file 4: Figure S3b). The LD level, based on R^2^ values calculated from 1.6 million SNPs (minor allele frequency [MAF] ≥ 0.05), was higher in temperate maize than in tropical maize (Fig. 4c, Additional file 4: Figure S3c), which is consistent with previous reports [30]. Further analyses of LD blocks revealed 55,793 and 44,231 LD blocks across chromosomes in temperate and tropical maize lines, respectively. The number of LD blocks ≥ 5 kb in size in temperate maize lines was twice that in tropical maize lines (Fig. 4e, Additional file 4: Figure S3e, Additional file 5: Figure S4). The background recombination rate predicted for each SNP interval in temperate and tropical maize populations based on the TE frequency indicated a higher background recombination rate in tropical maize than in temperate maize (2.157/kb versus 1.997/kb, Fig. 4d, Additional file 4: Figure S3d), indicating that temperate maize lines have a higher NRTE insertion frequency, along with lower BR rates and larger LD blocks, compared to tropical maize, which supports the findings of previous studies [5, 7]. However, the patterns of population differentiation associated with NRTE insertions under contrasting climates may simply reflect the population structure along environmental gradients [31].

### NRTEs affecting genes related to habitat adaptation

NRTEs with a frequency > 0.05 and insertion site within 1 kb upstream or downstream of genes were selected. The functions of genes adjacent to those harboring these NRTEs were manually examined. Among the 13,244 and 14,217 candidate genes in temperate and tropical maize, 238 and 217 genes potentially affected or regulated by upstream or downstream TEs were annotated as involved in flowering time-related traits and 13 categories of abiotic and biotic stresses in temperate and tropical maize lines (Additional file 6: Table S6), respectively, in blast2go [32]. In temperate lines, 171 genes near NRTEs were annotated as stress-related genes involved in defense response, heat shock, and wound response; and 67 genes associated with flowering time were found near the NRTEs, compared to 156 stress-related genes and 61 flowering-related genes near NRTEs, found in tropical lines. Among them, two NRTEs located near the genes related to senescence reduction and universal stress were only found in the tropical lines. Among TE classes, DNA TEs accounted for the highest proportion of TEs (60% in temperate and 56% in tropical lines) located near genes, which is in agreement with previous reports on the distribution pattern and insertion preferences of DNA TEs.

### Phenotypic variation affecting adaptation and GWAS of NRTEs

The most obvious and direct changes observed in tropical maize when grown in a temperate climate are delayed flowering, pollination and seed set, reduced grain-filling period, and increased grain moisture at harvest, which ultimately contribute to grain yield reduction [33, 34]. Therefore, photoperiod sensitivity and flowering-related traits are key characteristics for evaluation of the environmental adaptation of tropical maize to temperate conditions. In this study, seven phenotypic traits related to day length and photoperiod were collected from three locations (Beijing, Hainan, and Sichuan) (Additional file 6: Figure S5). Significant differences in flowering time were observed between temperate and tropical maize lines in each of the three locations, with temperate lines flowering significantly earlier than tropical lines (*p* < 0.01) (Additional file 7: Figure S6). Adaptive evolution involves the selection of different ecotypes, leading to measurable phenotypic differences between environments. To test for regional divergent selection, genetic differentiation based on F_st_ and Q_st_ values was computed from NRTE frequency and quantitative traits (Additional file 1: Table S7). The Q_st_-F_st_ method [35] made it possible to determine whether divergent traits resulted from directional selection (Q_st_ > F_st_), genetic drift (Q_st_ ~ F_st_), or stabilizing selection (Q_st_ < F_st_). Here, significant differences between temperate and tropical maize lines (*p* ≤ 2.78 × 10^−5^) resulting from directional selection (Q_st_ > F_st_) were found for DMF and DFF in the three locations and for EH in Sichuan.

**Fig. 5:**
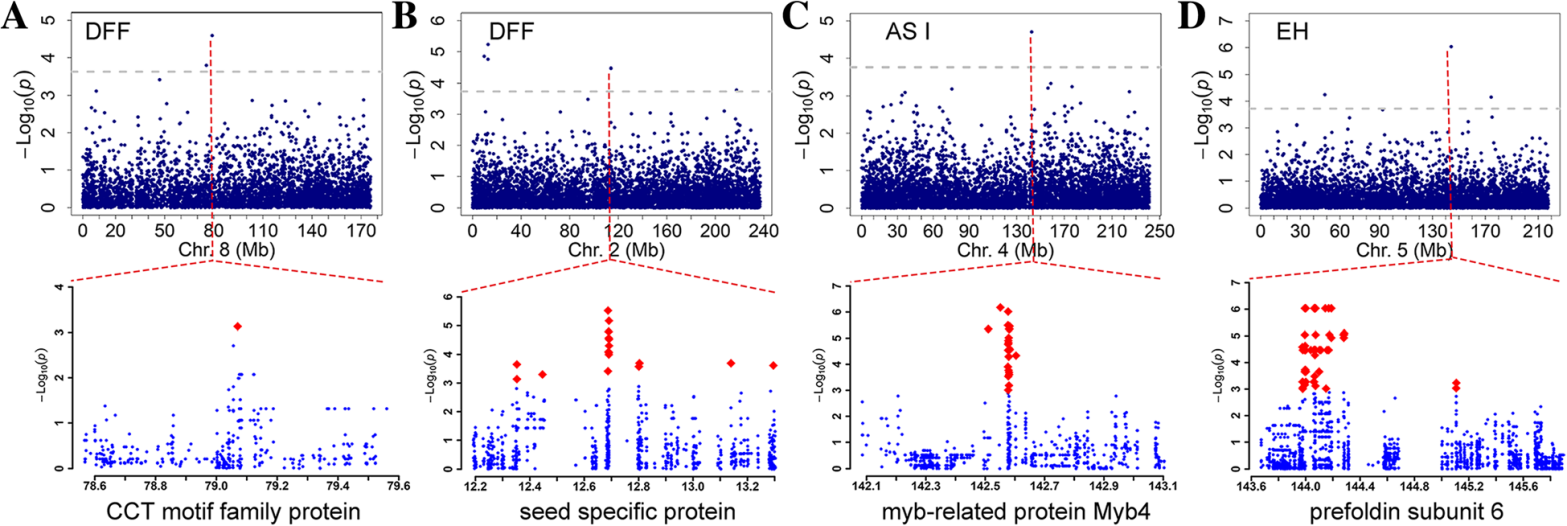
Genome-wide association study (GWAS) between non-redundant transposable elements (NRTEs) and flowering-related traits. The associated NRTE insertions (top) and the SNPs within the annotated gene (bottom) are marked by *red dotted lines* and *red dots*, respectively. **a** Days to female flowering (DFF) on chromosome 8, annotated as CCT motif family protein. **b** DFF on chromosome 2, annotated as seed specific protein. **c** Anthesis-silking interval (ASI) on chromosome 4, annotated as myb-related protein. **d** Ear length (EH) on chromosome 6, annotated as prefolding subunit 6

Although many TEs were detected near flowering-related genes, whether the NRTEs are involved in altering the regulation of these genes is unclear. Therefore, 48,296 NRTEs with MAF ≥ 0.15 were used for genome-wide association study (GWAS) of flowering traits. Among 48 NRTEs significantly associated with flowering traits at a Bonferroni-corrected threshold of *p* ≤ 2.07 × 10^−5^ (Additional file 1: Table S8), 38.3% were of the Gypsy superfamily, 25.5% were of the Copia superfamily, 31.9% were DNA TEs, and two NRTEs (4.2%) belonged to minor retrotransposon types. The genes harboring or adjacent to NRTEs included several known to be related to flowering time, such as the *CCT* motif family protein and myeloblastosis family transcription repressor *myb*4. GRMZM2G177400, which is involved in electron transfer reactions, was found near a LTR-RT/gypsy_uwum, and GRMZM2G039029 was found near a LTR-RT/gypsy_xilon, and both were located in regions associated with flowering date [5]. Association mapping was also performed to identify significant signals between flowering traits and SNPs located within 1 Mb upstream and 1 Mb downstream of candidate genes, harboring significant NRTEs to determine whether or not genetic variation within selected regions has contributed to the observed phenotypic changes. As a result, 10 candidate genes harbored significant SNPs associated with flowering-related traits (*p* < 0.001) (Fig. 5), suggesting that the candidate genes harboring NRTEs and SNPs could affect the phenotype of the targeted traits and NRTE-based makers identified more candidate sites than SNP makers (48 versus 10) by using the association mapping.

## Discussion

### Selection pressure revealed by joint analysis of NRTEs, LD, and recombination rates

The insertion of TE on genome affects the nearby gene expression, like the traditional genetic markers such as SSRs, InDels, and SNPs [5, 7], which have been employed for population structure and association analysis based on its insertion polymorphisms. In this study, a total of 274,408 NRTEs were identified from the 83 maize inbred lines, providing a valuable information additional to structural variations of genome. NRTEs were validated through a PCR-based analysis, and a high proportion (87%) of PCR validation in 83 lines showed true positive results, indicating the experimental validation agreed with the computational prediction from the resequencing data. The PCR validation of the transposons is relatively difficult because the primer designing usually is especially based on the sequences nearby the TE, which either has long insertion sequences or repetitive sequences. However, these four primers were used for a core maize collection with representative tropical and temperate germplasm, and the successful cases showed a high accuracy of NRTE detection among the 83 maize lines. However, a proportion (10%) of the NRTEs revealed by PCR were not detected by bioinformatics method because of the low depth of genome sequencing for the tested maize lines. Besides, there were 3% of the false positive cases in the genome scan of resequencing data. The false positives  may come from the copy number variations (CNVs) that existed in the tested maize but not in the B73 reference genome or from cross-contamination. They may have also resulted from residual heterozygosity or haplotype variation within the accession. Although there is a relatively high confidence for current NRTE validation with few primers, experimentation with more primers is still necessary for future research.

As reported previously, TEs, especially LTR-RTs, are preferentially located in gene-poor heterochromatic regions. This skewed distribution has often been interpreted as a consequence of preferential TE accumulation in recombination-suppressed regions, possibly as a result of reduced purifying selection against deleterious insertions relative to regions with high rates of recombination [24, 36]. In the present study, temperate maize was found to have 1.26 times more LD blocks than tropical maize; LD blocks with size ≥ 5 kb in temperate maize were two times more common than in tropical maize, which is consistent with a previous report [30]. In addition, the higher TE densities and lower TE frequencies found in tropical maize suggest that tropical maize contains more rare alleles. This significant difference appears to be the result of tropical maize experiencing a more intensive recombination [30, 37], which is supported by the higher BR rate found in tropical maize. Maize lines expanding to temperate environments experienced high selection pressure for traits that provided adaption to differing growing conditions, including changes in photoperiod and exposure to chilling [38]. Strong selection pressure might have reduced genetic diversity in temperate maize, resulting in low TE density and increased frequency of the remaining TEs.

Temperate maize lines showed higher TE frequency despite their lower TE number, which might be related to selective pressure and population bottleneck associated with the diffusion of maize into temperate climates. The expansion of maize cultivation from tropical to temperate regions required a post-domestication adaptation to certain climates, likely at least partially through changes in gene regulation. As reported previously, TEs located near genes may affect the expression of those genes through several mechanisms, including insertion of new cis-regulatory regions and effects on the chromatin state of gene promoter regions, leading to changes in how genes are regulated in response to environmental stimuli or stresses [39]. TEs within conserved non-coding sequences can cause dramatic functional variation that might function as responses and adaptions to environmental changes [20]. Therefore, a subset of transposon insertions that are present at high frequency in temperate maize and low or zero frequency in tropical maize may have been involved in conveying adaptive benefits in this new environment. Indeed, TEs are often targets of selection during evolution; the insertion of a Hopscotch retrotransposon upstream of the domestication gene *tb1* was shown to increase apical dominance in maize [19, 40], and insertion of the Rider retrotransposon increased the expression of *SUN*, leading to an elongated fruit shape in tomato [41].

### Environmentally-related, TE-induced adaptive evolution

In the past several thousand years, maize spread rapidly and adapted to a wide range of environments from 30° N to 48° S [1], resulting in significant phenotypic changes between ancestral tropical maize populations and varieties that grow in temperate regions [20] . Adaptive evolution is considered to play an important role in determining the fate of maize in response to different environmental factors such as photoperiod and temperature [42] . TEs are likely to play an important role in adaptive evolution because of their ability to generate mutations and be involved in gene regulation [12] . A recent report showed that many TEs contribute to activation of maize genes in response to abiotic stress [39]. Our results also suggest that many NRTEs are different between temperate and tropical maize lines and that several NRTEs are related to flowering traits, which might contribute to the adaptation of maize to longer photoperiods.

Similar to the present study [19–21], many of the NRTEs identified recently are located either within or adjacent to particular genes. Some of these inserted TEs likely modify the transcriptional regulation of their associated genes. GWAS and annotation analyses in this study indicated that the presence or absence of NRTEs near flowering-related genes could be associated with observed variation in flowering time in the same maize lines. This suggests that changes in transcriptional regulation, induced by the insertion of transposons into gene promoters, may have played an important role in the adaptation to major variations in associated temperature and photoperiod, which is correlated with the rapid dissemination of maize over a large part of the globe over a period of only a few thousand to a few hundred years.

## Conclusions

In this study, genome-wide polymorphic transposon insertion sites were identified using resequencing data in a panel of diverse maize lines, representing temperate and tropical germplasm. A comparative analysis of NRTEs, recombination rate, and linkage disequilibrium in temperate and tropical maize revealed that selection on NRTEs may have contributed to the adaptation of maize to temperate environments. Compared to tropical maize, temperate maize had fewer unique NRTEs but a higher insertion frequency, lower background recombination rates, and higher level of linkage disequilibrium, with more NRTEs found close to flowering and stress-related genes. Association mapping demonstrated that presence/absence variation for 48 NRTEs was associated with variation in flowering-time phenotypes, as well as variation in the expression of genes neighboring the transposon insertions. This study suggests that TEs may have played an important role in enabling maize to adapt to different climates following domestication.

## Methods

### Prediction and validation of NRTE insertions in tropical and temperate maize

The resequencing data were generated from 83 maize inbred lines (Additional file 1: Table S1) obtained from China, the United States, and the International Maize and Wheat Improvement Center (CIMMYT) based in Mexico, Kenya, and Zimbabwe. According to their origin and geographical distribution, the maize lines were classified to 31 temperate and 52 tropical lines that had an average sequencing depth of 13.21× and 14.12×, respectively; 87.88% of the temperate maize reads and 85.03% of the tropical maize reads were mapped to the B73_AGPv2 reference genome (http://www.maizegdb.org). The sequencing reads were included in the HapMap2, which were retrieved from the Sequence Read Archive (SRP011907, http://www.ncbi.nlm.nih.gov/sra/) [43] .

Following Tian et al. [24] and Ewing and Kazazian [25], a semi-automated bioinformatics pipeline was developed to identify the NRTE insertions present in the resequenced reads of the 83 maize lines. NRTEs were identified based on the 20–55 bp TE junction sequences since the chance that two inserting TEs shared >20 bp flanking sequences in the same genome would be really low (< 4^−20^). Firstly, a TE database comprising complete maize sequences was downloaded from the Maize database (http://maizetedb.org/~maize/) [26]. For each of the 1526 TE sequences, the 100 base pairs at each end of the sequence were selected and combined into a database of TE edge sequences. After quality control, including removing the adapters, filtering/trimming back-trailing bases of low quality, and filtering short reads, the remaining high-quality reads from the 83 maize lines were trimmed to 75 base pairs and aligned to the TE edge sequence library. The reads, which had 20–55 bp perfectly (100%) aligned to the extremity of TE-edge fragments, were considered to contain the TE insertion junction sites and were kept for further analysis. These reads were then mapped to the maize B73_AGPv2 reference genome [22]. To avoid the potential ascertainment bias due to using the B73 (temperate) reference genome to characterize temperate versus tropical NRTEs, the reads containing at least 65 bp with at least 95% sequence identity match to the reference genome were considered to contain TE insertion sites and were excluded from further analyses. Reads showing a 20–55 bp (TE franking sequences) perfect match to the reference genome were considered to contain NRTE insertions. The number and frequency of NRTE insertions, and the TE class they belong to, were computed for temperate and tropical maize lines.

To validate the reliability of the pipeline used to predict NRTEs, a polymerase chain reaction (PCR)-based scoring method was used. Four NRTE insertion sites located near genes and showing different frequencies in the resequencing-based analysis of temperate and tropical maize were selected for validation using 75 resequenced maize lines (Additional file 1: Table S1). Primers were designed based on the flanking sequences of NRTEs (Additional file 1: Table S4). The touchdown PCR reaction was performed under the following conditions: pre-denaturation at 95 °C for 5 min; 6 cycles of denaturation at 95 °C for 40 s, annealing at 59 °C for 30 s, and extension at 72 °C for 1 min; 33 cycles of denaturation at 95 °C for 30 s, annealing at 54 °C for 30 s, and extension at 72 °C for 1 min; and a final extension at 72 °C for 10 min. PCR products were sequenced and searched for homology against the National Center for Biotechnology Information (NCBI) nucleotide library.

### TE scanning and estimation of GR rates in the B73 reference genome

The TEs present in the B73 reference genome were scanned using RepeatMasker 4.0.5 (http://www.repeatmasker.org) [44] with default parameters. Simple sequence repeats (SSRs) and the genetic map of the intermated B73 × Mo17 (IBM2) population were downloaded from MaizeGDB [28, 29], and used to calculate GR rates in MareyMap, a statistical package in R [45] . Pericentromeric regions were defined based on [46], where the modified transposon display method was used to screen a large number of potential centromeric markers polymorphic between the two parents of the mapping population [46].

### Correlation of NRTE distributions and genomic features in temperate and tropical maize

To evaluate whether or not the distribution of NRTE insertions in temperate and tropical maize lines is associated with gene densities and GR rates, each chromosome was divided into contiguous 1-Mb regions (called windows). The gene distribution within each 1-Mb contiguous window along each chromosome was obtained from the ZmB73_5b_FGS maize genome (ftp://ftp.gramene.org/pub/maize). The proportion of NRTEs and gene densities within each window were calculated for each maize line. Tendencies in NRTE distribution were assessed through correlation analysis of NRTE insertions in temperate and tropical maize with the accumulated TEs, gene densities, and GR rates in the B73 genome, using Pearson’s correlation in the R-project software package [47] . NRTE densities in chromosome arms and pericentromeric regions and the number of NRTEs in UTR, introns, and exons were calculated using Perl scripts. In addition, 1.6 million SNPs (MAF ≥ 0.05) generated from the 83 resequencing genomes were used to estimate the LD patterns in temperate and tropical maize, respectively. SNPs were separated into two groups, according to the polymorphisms in temperate and tropical maize, and tested for differences using Haploview v3.0 [48, 49], with the following parameters: minGeno: 0.5, hwcutoff: 0.001, max Distance: 500, missing Cutoff: 0.5, check, and dprime. LD blocks were also predicted in Haploview by using the block output option GAB [50] . The background recombination (BR) rate was predicted for each SNP interval in temperate and tropical maize populations based on TE insertions by using Phase v 2.1.1, with the default parameters [51].

### Analysis of directional selection on NRTEs and phenotypic divergence

Seven phenotypic traits from the 83 maize lines including days to male flowering, days to female flowering, anthesis-silk interval, plant height, ear height, tassel length, and tassel branch number were collected in three locations (Beijing, Hainan, and Sichuan) which have different day lengths (Additional file 6: Figure S5), each with two replicates. For complex polygenic traits, Q_st_-F_st_ comparative studies on the divergence of quantitative traits and neutral molecular markers were used to distinguish between natural selection and genetic drift as the main cause of population differentiation [52]. F_st_ was calculated based on NRTEs and Q_st_ was estimated for all phenotypic traits (Additional file 1: Table S7) [52, 53]. Q_st_ is defined as the total genetic variation in the trait and represented as Q_st_ = σ^2^_GB_ / (σ^2^_GB_ + 2σ^2^_GW_), where σ^2^_GB_ is the variance component among population and σ^2^_GW_ is that within population. To accurately estimate the distribution of mean F_st_ in tropical and temperate lines, 1000 NRTE insertions were randomly chosen from the entire dataset, and the calculation was repeated 1000 times. Applying a strict outlier definition, a 99% confidence interval was used for F_st_ distribution, ensuring a more accurate comparison between Q_st_ and F_st_. This enabled us to determine whether or not the divergent traits are the result of directional selection (Q_st_ > F_st_), genetic drift (Q_st_ ≈ F_st_), or stabilizing selection (Q_st_ < F_st_) [52].

### NRTE-based GWAS for flowering-related traits and gene expression

The 48,296 NRTE insertions regarded as markers (MAF ≥ 0.15) were selected for GWAS. A general linear model (GLM) that accounted for population structure was performed using TASSEL 3.0 [54] to test for associations between the seven flowering-related traits and NRTE insertions. A Bonferroni-adjusted significance threshold of *p* ≤ 3.33 × 10^−5^ was used to identify significant associations. A principal component analysis (PCA) was performed using the GAPIT package of R-project [55], based on 30,000 randomly selected SNPs from the total 1.8 million SNPs called from the 83 resequenced maize inbred lines. SNPs located between 1 Mb upstream and 1 Mb downstream of the genes harboring or nearby NRTE insertions related to flowering traits were extracted, and the significant associations between these SNPs and flowering traits were evaluated through GLM, using a *p* < 0.001 as cutoff to identify significant associations, since the number of SNPs in each test is usually hundreds. Manhattan plots were obtained for *p* values in R-project. In addition, association mapping was also performed on NRTE *DTM-4-225,713,639* with the expression of 28,850 genes in 368 maize inbreds [27] to reveal the impact of this NRTE on gene regulation, at a uniform Bonferroni-adjusted significance threshold of *p* ≤ 3.47 × 10^−5^ (α = 1).

## Additional files

Additional file 1: Table S1. Information of the 83 maize inbred lines. **Table S2.** Gene sequences harboring non-redundant transposable elements (NRTEs) in the 83 maize inbred lines. **Table S3.** Distribution of non-redundant transposable elements (NRTEs) in chromosomal arms and pericentromeric regions across 10 chromosomes. **Table S4.** Primers used for validation of non-redundant transposable elements (NRTEs). **Table S5.** Correlation of accumulated transposable element (TE) contents with genomic features in the reference genome. **Table S6.** Stress- and flowering-related genes and their adjacent non-redundant transposable elements (NRTEs). **Table S7.** Traits showing significant divergence and directional selection patterns in temperate and tropical maize lines. **Table S8.** Genome-wide association study of flowering-related traits and non-redundant transposable elements (NRTEs). (XLSX 47 kb)

Additional file 2: Figure S1. Gene sequences harboring non-redundant transposable elements (NRTEs) in the 83 maize inbred lines. (a) Gene sequences harboring LTR_RTs in the 83 maize inbred lines. (b) Gene sequences harboring DNA TEs in the 83 maize inbred lines. (TIFF 133 kb)

Additional file 3: Figure S2. Characterization of transposable element (TE) distribution and genomic features along chromosomes 2–10. (a) Distribution of accumulated TEs and genes in the B73 reference genome. (b-c) Distribution of non-redundant TEs (NRTEs) in the temperate and tropical maize lines. (d) Variation of genetic recombination (GR) rates along the chromosome in the B73 reference genome. The pink highlighted area defines the pericentromeric region on the chromosome. cM: centimorgans. (TIFF 5649 kb)

Additional file 4: Figure S3. Relationship among NRTEs, BR rates, and LD patterns in temperate and tropical maize on the chromosomes 2–10. (a-b) Frequencies and densities of NRTEs in temperate and tropical maize lines. (c-d) LD patterns revealed by R^2^ and BR rate in temperate and tropical maize lines. (e) Distribution differences of LD blocks between temperate and tropical maize lines. Green and red boxes indicate LD blocks (> 5 kb) in temperate and tropical maize lines, respectively. NRTE: non-redundant transposable element; LD: linkage disequilibrium; BR: background recombination. (TIFF 4040 kb)

Additional file 5: Figure S4. Statistics of LD blocks between temperate and tropical maize lines. (a) Overview of LD block size distribution. (b-c) Statistics for LD blocks with sizes of ≤ 100 bp and ≥ 20 kb, respectively. (TIFF 376 kb)

Additional file 6: Figure S5. Statistics of flowering-related traits in tropical and temperate maize lines. (TIFF 191 kb)

Additional file 7: Figure S6. Simulation of the distribution of F_st_ mean values between temperate and tropical maize lines. (TIFF 154 kb)

## Abbreviations

ASI: Anthesis-silk interval
BJ: Beijing
BR: Background recombination
CIMMYT: International Maize and Wheat Improvement Center
CNVs: Copy number variations
DFF: Days to female flowering
DMF: Male flowering
EH: Ear height
GLM: General linear model
GR: Genetic recombination
GWAS: Genome-wide association study
HN: Hainan
IBM2: Intermated B73 × Mo17
LD: Linkage disequilibrium
LTR-RTs: Long terminal repeat retrotransposons
MAF: Minor allele frequency
NCBI: National Center for Biotechnology Information
NRTEs: Non-redundant TEs
PCA: Principal component analysis
PCR: Polymerase chain reaction
PH: Plant height
SC: Sichuan
SSRs: Simple sequence repeats
TBN: Tassel branch number
TE: Transposable element
TL: Tassel length
UTRs: Untranslated regions
