# Supplementary figures and images for: Genome-wide characterization of non-reference transposable element insertion polymorphisms reveals genetic diversity in tropical and temperate maize
Source: BMC Genomics. 2017 Sep 6;18:702. doi: 10.1186/s12864-017-4103-x (PMC5588714; doi:10.1186/s12864-017-4103-x)

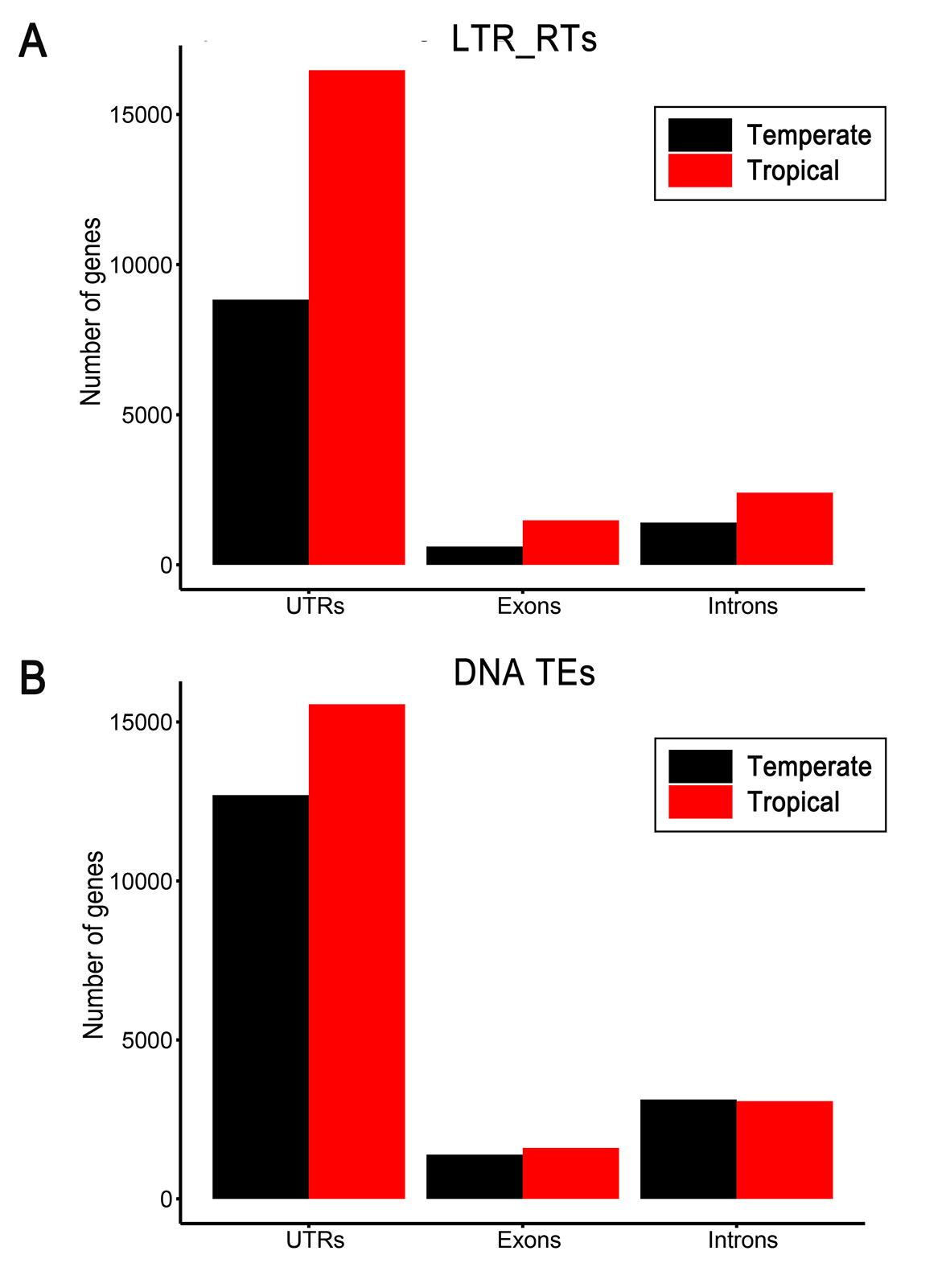

Supplement: Supplementary file 2 — Gene sequences harboring non-redundant transposable elements (NRTEs) in the 83 maize inbred lines. (a) Gene sequences harboring LTR_RTs in the 83 maize inbred lines. (b) Gene sequences harboring DNA TEs in the 83 maize inbred lines. (TIFF 133 kb) [file 12864_2017_4103_MOESM2_ESM.tif]

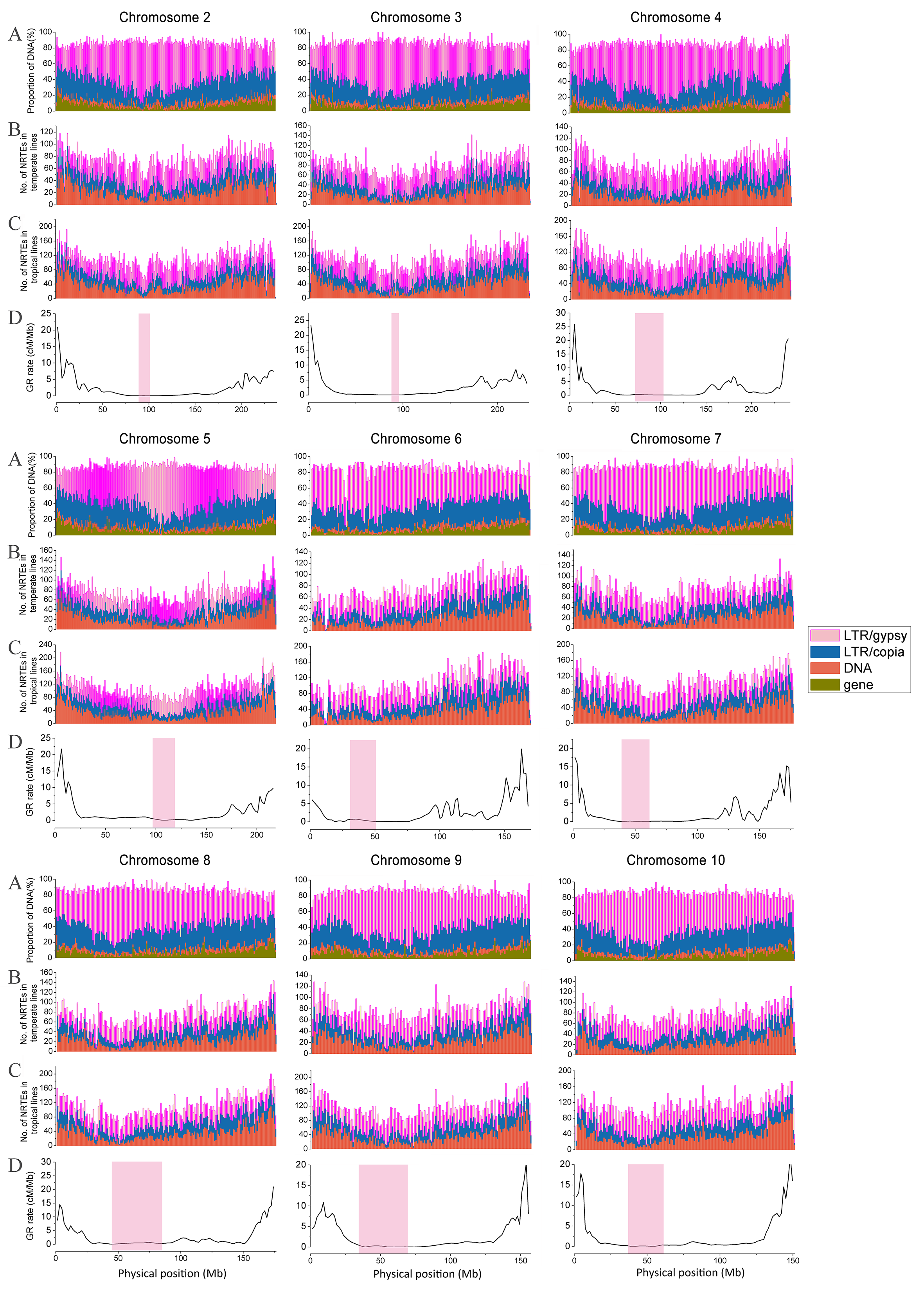

Supplement: Supplementary file 3 — Characterization of transposable element (TE) distribution and genomic features along chromosomes 2–10. (a) Distribution of accumulated TEs and genes in the B73 reference genome. (b-c) Distribution of non-redundant TEs (NRTEs) in the temperate and tropical maize lines. (d) Variation of genetic recombination (GR) rates along the chromosome in the B73 reference genome. The pink highlighted area defines the pericentromeric region on the chromosome. cM: centimorgans. (TIFF 5649 kb) [file 12864_2017_4103_MOESM3_ESM.tif]

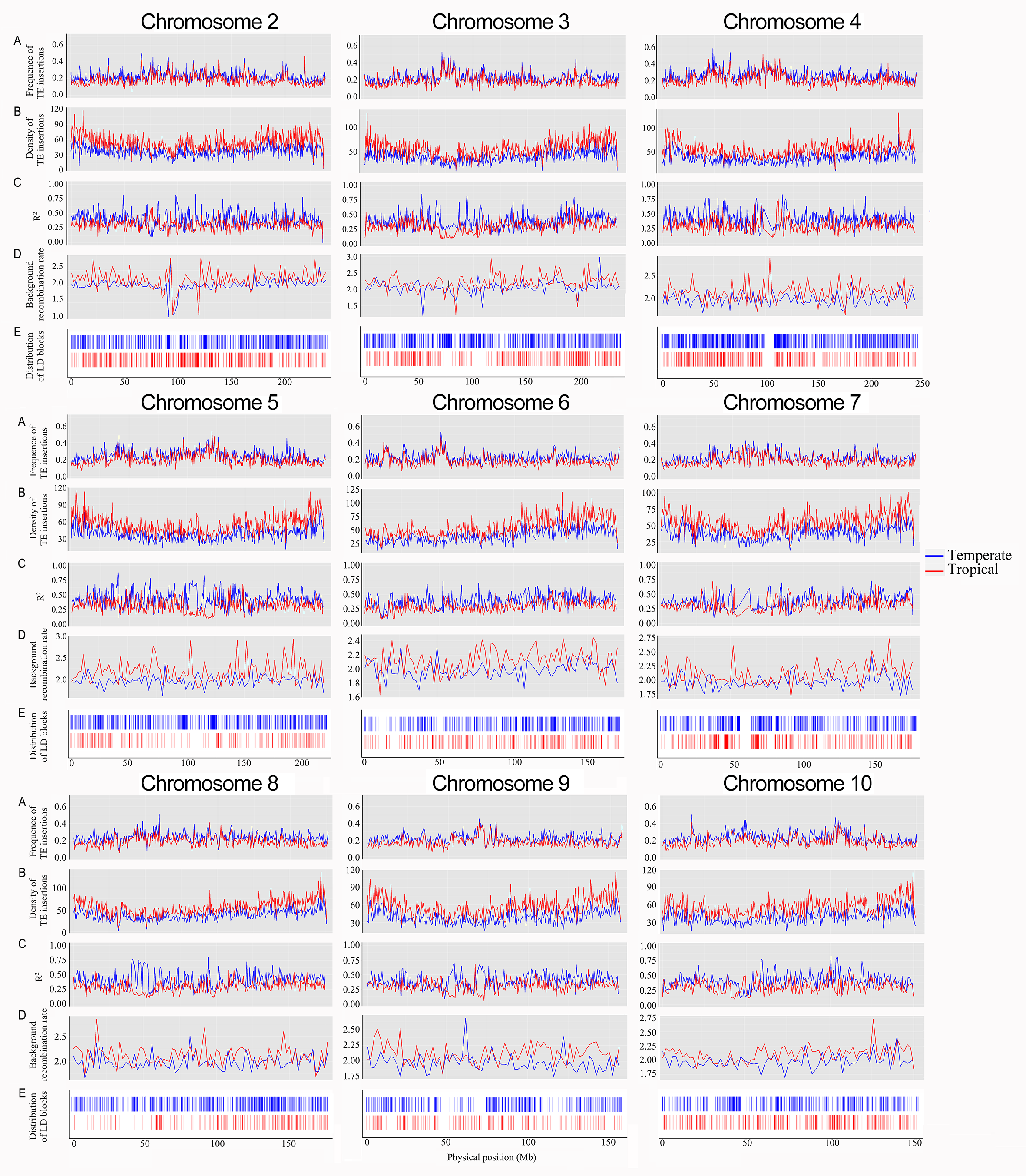

Supplement: Supplementary file 4 — Relationship among NRTEs, BR rates, and LD patterns in temperate and tropical maize on the chromosomes 2–10. (a-b) Frequencies and densities of NRTEs in temperate and tropical maize lines. (c-d) LD patterns revealed by R2 and BR rate in temperate and tropical maize lines. (e) Distribution differences of LD blocks between temperate and tropical maize lines. Green and red boxes indicate LD blocks (> 5 kb) in temperate and tropical maize lines, respectively. NRTE: non-redundant transposable element; LD: linkage disequilibrium; BR: background recombination. (TIFF 4040 kb) [file 12864_2017_4103_MOESM4_ESM.tif]

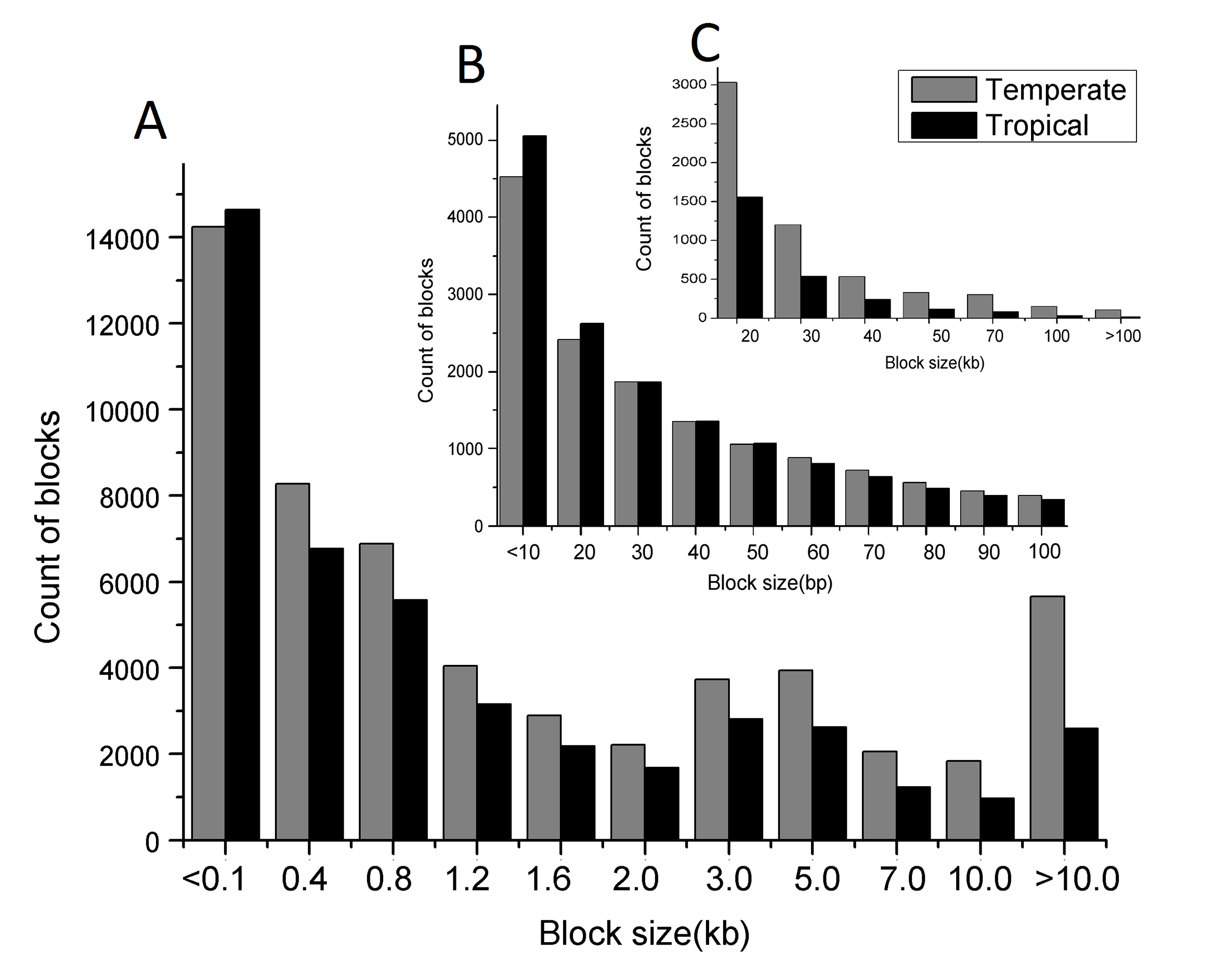

Supplement: Supplementary file 5 — Statistics of LD blocks between temperate and tropical maize lines. (a) Overview of LD block size distribution. (b-c) Statistics for LD blocks with sizes of ≤ 100 bp and ≥ 20 kb, respectively. (TIFF 376 kb) [file 12864_2017_4103_MOESM5_ESM.tif]

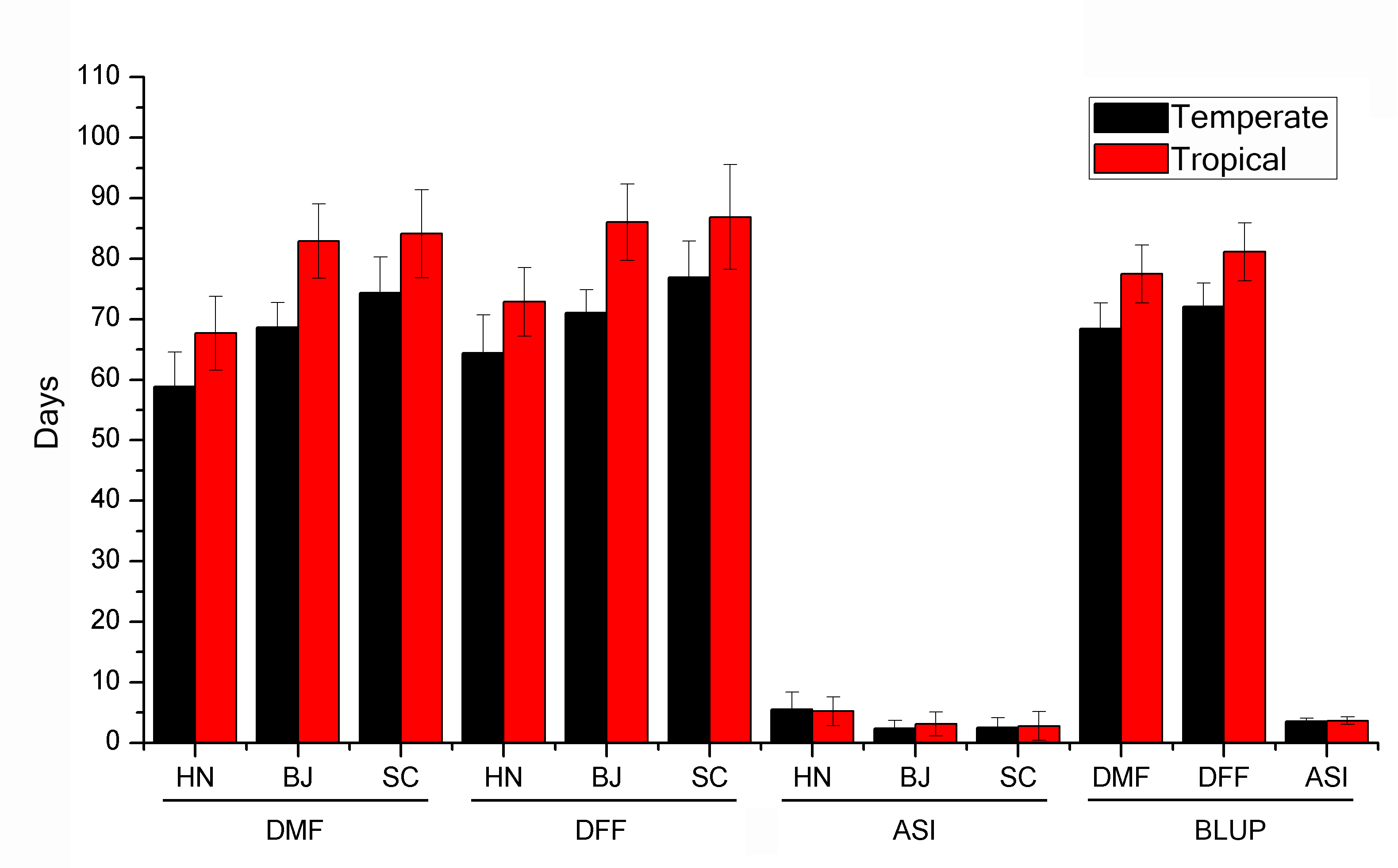

Supplement: Supplementary file 6 — Statistics of flowering-related traits in tropical and temperate maize lines. (TIFF 191 kb) [file 12864_2017_4103_MOESM6_ESM.tif]

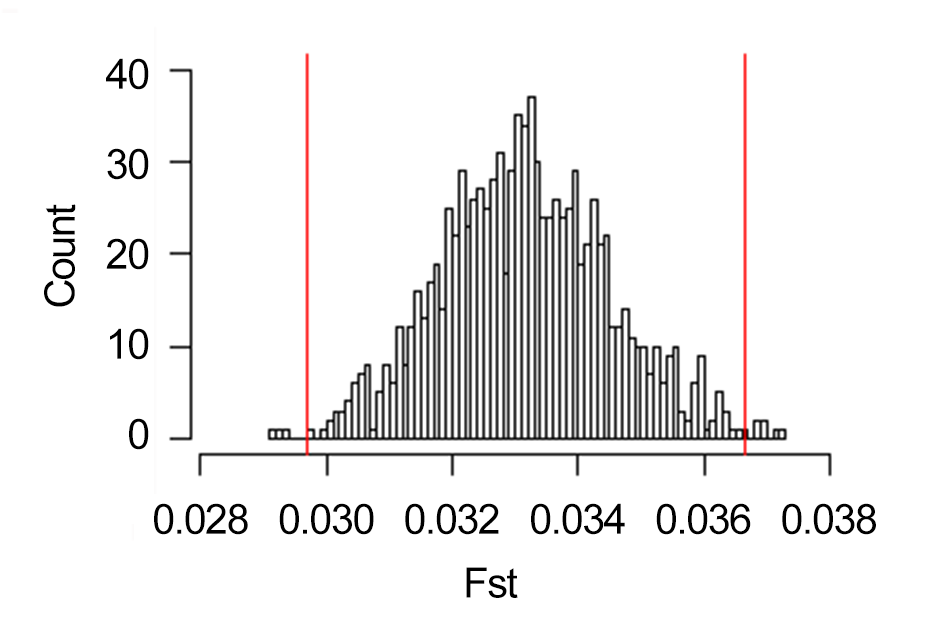

Supplement: Supplementary file 7 — Simulation of the distribution of Fst mean values between temperate and tropical maize lines. (TIFF 154 kb) [file 12864_2017_4103_MOESM7_ESM.tif]
